# Diazido­bis(5,5′-dimethyl-2,2′-bipyridyl-κ^2^ *N*,*N*′)nickel(II) monohydrate

**DOI:** 10.1107/S1600536809029407

**Published:** 2009-08-08

**Authors:** Jaturong Phatchimkun, Palangpon Kongsaeree, Nattawut Suchaichit, Narongsak Chaichit

**Affiliations:** a Department of Computer Science, Faculty of Engineering, Vongchavalitkul University, Nakornrachasima 30000, Thailand; b Department of Chemistry, Faculty of Science, Mahidol University, Bangkok 10400, Thailand; c Department of Applied Chemistry, Faculty of Science and Liberal Arts, Rajamangala University of Technology Isan, Nakornrachasima 30000, Thailand; d Department of Physics, Faculty of Science and Technology, Thammasat University, Rangsit, Pathumthani 12121, Thailand

## Abstract

In the crystal structure of the title compound, [Ni(N_3_)_2_(C_12_H_12_N_2_)_2_]·H_2_O, the Ni^II^ atom is situated on a twofold axis and adopts a distorted octa­hedral geometry with the two 5,5′-dimethyl-2,2′-bipyridyl (dmbpy) and the two azide ligands in a *cis* arrangement. The water solvent mol­ecule is disordered over two positions in a 1:1 ratio.

## Related literature

For general background to 2,2′-bipyridine and its derivatives, see: Blau (1888 ▶); Constable (1989 ▶); Constable & Steel (1989 ▶); Juris *et al.* (1988 ▶). For related dmbpy structures, see: van Albada *et al.* (2004 ▶, 2005 ▶); Catalan *et al.* (1995 ▶); Kooijman *et al.* (2002 ▶). For Ni–N bond lengths in azido-containing mononuclear nickel(II) complexes, see: Urtiaga *et al.* (1995 ▶). For an Ni^II^ complex with 5,5′-dimethyl-2,2′-bipyridyl, see: Hou (2008 ▶). For a description of the Cambridge Structural Database, see: Allen (2002 ▶).
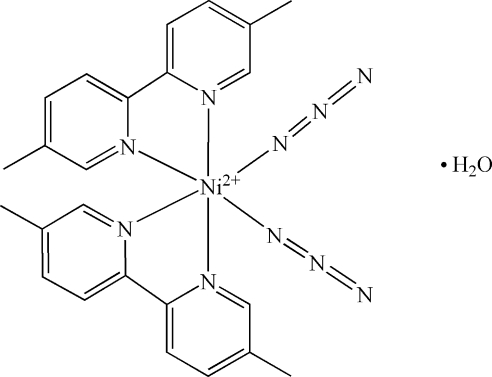


## Experimental

### 

#### Crystal data

[Ni(N_3_)_2_(C_12_H_12_N_2_)_2_]·H_2_O
*M* *_r_* = 529.22
Orthorhombic, 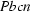

*a* = 17.0770 (3) Å
*b* = 8.5350 (5) Å
*c* = 16.6700 (4) Å
*V* = 2429.69 (16) Å^3^
*Z* = 4
Mo *K*α radiation
μ = 0.84 mm^−1^
*T* = 293 K
0.53 × 0.45 × 0.40 mm

#### Data collection

Nonius KappaCCD diffractometer
Absorption correction: multi-scan (*SADABS*; Sheldrick, 1996 ▶) *T*_min_ = 0.630, *T*_max_ = 0.712
7908 measured reflections
4209 independent reflections
2929 reflections with *I* > 2σ(*I*)
*R*_int_ = 0.031

#### Refinement

*R*[*F* ^2^ > 2σ(*F* ^2^)] = 0.043
*wR*(*F* ^2^) = 0.127
*S* = 1.03
4209 reflections
195 parameters
H atoms treated by a mixture of independent and constrained refinement
Δρ_max_ = 0.35 e Å^−3^
Δρ_min_ = −0.37 e Å^−3^

### 

Data collection: *COLLECT* (Nonius, 2002 ▶); cell refinement: *COLLECT* and *DENZO*/*SCALEPACK* (Otwinowski & Minor, 1997 ▶); data reduction: *COLLECT*; program(s) used to solve structure: *SHELXS97* (Sheldrick, 2008 ▶); program(s) used to refine structure: *SHELXL97* (Sheldrick, 2008 ▶); molecular graphics: *SHELXTL* (Sheldrick, 2008 ▶); software used to prepare material for publication: *SHELXTL*.

## Supplementary Material

Crystal structure: contains datablocks I, global. DOI: 10.1107/S1600536809029407/rn2055sup1.cif

Structure factors: contains datablocks I. DOI: 10.1107/S1600536809029407/rn2055Isup2.hkl

Additional supplementary materials:  crystallographic information; 3D view; checkCIF report

## Figures and Tables

**Table 1:** Selected geometric parameters (Å, °)

Ni1—N1	2.0882 (13)
Ni1—N2	2.0897 (13)
Ni1—N3	2.1053 (14)

Symmetry code: (i) 
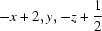
.

**Fig. 1.**
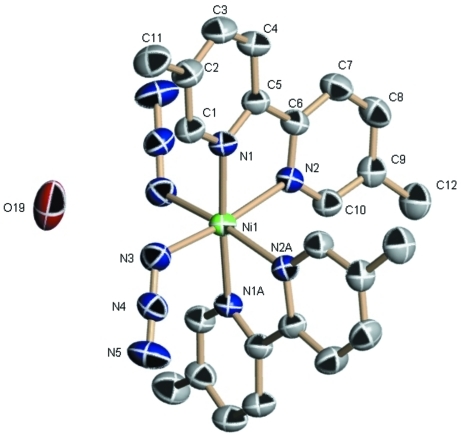
A view of the title structure with the atom-numbering scheme and displacement ellipsoids drawn at the 30% probability level. H atoms have been omitted for clarity.

## supplementary crystallographic information

### Comment

It is 121 years since Blau first reported (Blau, 1888) the synthesis of 2,2'-bipyridine (bpy) and described the first metal complexes of this important ligand. Since then bpy and its derivatives have been continuously and extensively used in several aspects of coordination chemistry (Constable, 1989; Constable & Steel, 1989; Juris *et al.*, 1988). However, up to now comparatively few X-ray crystal structures of ligand 5,5'-dimethyl-2,2'-bipyridyl (dmbpy) have been published (Albada *et al.*, 2004; Catalan *et al.*, 1995; Kooijman *et al.*, 2002). In this study, we report the synthesis and characterization of the new Ni(II) complex containing both dmbpy and azido ligands. Although 52 structures containing dmbpy with metals were found in the Cambridge Structural Database (CSD; Version 5.29, November 2008 update; Allen, 2002) there was only one case (CSD refcode POMFAZ; Hou, 2008) where en is present with both Ni^II^ and 5,5'-dimethyl-2,2'-bipyridyl.

It is found that the Ni ion is coordinated by four nitrogen atoms from dmbpy and two azido nitrogen atoms, taking on a distorted octahedral geometry. The two bidentate ligands have a *cis* disposition around the metal ion, forming practically perpendicular planes [N2—Ni1—N2^i^ 89.80 (7), N1—Ni1—N1^i^ 176.19 (7)°] (symmetry codes: i -*x* + 2, *y*, -*z* + 1/2). The rigidity of these ligands causes the bond angles N1— Ni1—N2, 78.26 (5)° to deviate significantly from orthogonality. This causes the geometry about the Ni^II^ ion to deviate slightly from that of an ideal octahedron. The Ni(1)—N(dmbpy) bond distances in a related complex [2.0897 (13) and 2.0882 (13) Å] (Urtiaga *et al.*, 1995) are almost the same as those found in the Ni(II) compound of [Ni^II^(bpy)_2_(N_3_)_2_] [2.067 (2)–2.114 (2) Å]. Good agreement is observed between the Ni(1)–*N*(azido) bond distance of 2.1053 (14) Å and those reported [2.094 (2) and 2.102 (3) Å] (Urtiaga *et al.*, 1995) for azido containing mononuclear nickel(II) complexes.

### Experimental

All chemicals were obtained commercially and used without further purification. The compound was prepared by adding a warm solution of dmbpy (0.181 g, 1.0 mmol) in methanol (15 ml) to a warming aqueous solution (10 ml) of Ni(CH_3_COO)_2_. 4H_2_O (0.123 g, 0.5 mmol). Afterward a solid of NaN_3_ (0.065 g, 1.0 mmol) was added. The green solution was slowly evaporated at room temperature and after few days, green plate-shaped crystals of [Ni(dmbpy)_2_(N_3_)_2_].H_2_O formed. They were filtered off, washed with mother liquid and air-dried. Yield *ca* 89%. Elemental analysis (%) found (calculated): C 54.4 (54.6), H 4.9 (4.8), N 26.3 (26.5). The IR spectrum shows the band corresponding to the asymmetric stretch of the azido ion, *ν*_as_(N_3_), split at 2072 and 2024 cm^-1^. This indicates that the azido ligand is bonded asymmetrically by its two terminal N atoms.

### Refinement

The water O atom is disordered which site occupancies of 0.5 and 0.5. All non-H atoms were refined anisotropically. H atoms in aromatic were placed in idealized positions and constrained to ride on their parent atoms, with C—H distances of 0.96–1.01 Å [*U*_iso_ (H) = 1.2Ueq (C)]. H atoms of methyl groups of dmbpy were placed in calculated positions and refined with a riding model C—H = 0.96 Å.

### Crystal data

**Table:**

[Ni(N_3_)_2_(C_12_H_12_N_2_)_2_]·H_2_O	*F*(000) = 1096
*M**_r_* = 529.22	*D*_x_ = 1.444 Mg m^−^^3^*D*_m_ = 1.440 Mg m^−^^3^*D*_m_ measured by flotation in aqueous KI
Orthorhombic, *P**b**c**n*	Mo *K*α radiation, λ = 0.71073 Å
Hall symbol: -P 2n 2ab	Cell parameters from 7908 reflections
*a* = 17.0770 (3) Å	θ = 2.7–27.5°
*b* = 8.5350 (5) Å	µ = 0.84 mm^−^^1^
*c* = 16.6700 (4) Å	*T* = 293 K
*V* = 2429.69 (16) Å^3^	Plate, green
*Z* = 4	0.53 × 0.45 × 0.40 mm

### Data collection

**Table:**

Nonius KappaCCD diffractometer	4209 independent reflections
Radiation source: fine-focus sealed tube	2929 reflections with *I* > 2σ(*I*)
graphite	*R*_int_ = 0.031
ω scans	θ_max_ = 32.0°, θ_min_ = 2.7°
Absorption correction: multi-scan (*SADABS*; Sheldrick, 1996)	*h* = −25→25
*T*_min_ = 0.630, *T*_max_ = 0.712	*k* = −12→12
7908 measured reflections	*l* = −24→24

### Refinement

**Table:**

Refinement on *F*^2^	Secondary atom site location: difference Fourier map
Least-squares matrix: full	Hydrogen site location: inferred from neighbouring sites
*R*[*F*^2^ > 2σ(*F*^2^)] = 0.043	H atoms treated by a mixture of independent and constrained refinement
*wR*(*F*^2^) = 0.127	*w* = 1/[σ^2^(*F*_o_^2^) + (0.0689*P*)^2^ + 0.3234*P*] where *P* = (*F*_o_^2^ + 2*F*_c_^2^)/3
*S* = 1.03	(Δ/σ)_max_ < 0.001
4209 reflections	Δρ_max_ = 0.35 e Å^−^^3^
195 parameters	Δρ_min_ = −0.37 e Å^−^^3^
0 restraints	Extinction correction: *SHELXL97* (Sheldrick, 2008), Fc^*^=kFc[1+0.001xFc^2^λ^3^/sin(2θ)]^-1/4^
Primary atom site location: structure-invariant direct methods	Extinction coefficient: 0.023 (3)

### Special details

**Table:**

Geometry. All e.s.d.'s (except the e.s.d. in the dihedral angle between two l.s. planes) are estimated using the full covariance matrix. The cell e.s.d.'s are taken into account individually in the estimation of e.s.d.'s in distances, angles and torsion angles; correlations between e.s.d.'s in cell parameters are only used when they are defined by crystal symmetry. An approximate (isotropic) treatment of cell e.s.d.'s is used for estimating e.s.d.'s involving l.s. planes.
Refinement. Refinement of *F*^2^ against ALL reflections. The weighted *R*-factor *wR* and goodness of fit *S* are based on *F*^2^, conventional *R*-factors *R* are based on *F*, with *F* set to zero for negative *F*^2^. The threshold expression of *F*^2^ > σ(*F*^2^) is used only for calculating *R*-factors(gt) *etc*. and is not relevant to the choice of reflections for refinement. *R*-factors based on *F*^2^ are statistically about twice as large as those based on *F*, and *R*- factors based on ALL data will be even larger.

### Fractional atomic coordinates and isotropic or equivalent isotropic displacement parameters (Å^2^)

**Table:**

	*x*	*y*	*z*	*U*_iso_*/*U*_eq_	Occ. (<1)
Ni1	1.0000	0.25758 (3)	0.2500	0.03045 (11)	
C1	0.85037 (9)	0.1839 (2)	0.15504 (10)	0.0394 (4)	
C2	0.77052 (9)	0.1813 (2)	0.13931 (10)	0.0430 (4)	
C3	0.72270 (11)	0.2632 (2)	0.19237 (13)	0.0485 (5)	
C4	0.75485 (10)	0.3478 (3)	0.25456 (10)	0.0444 (4)	
C5	0.83592 (9)	0.3501 (2)	0.26388 (9)	0.0351 (3)	
C6	0.87691 (8)	0.44736 (19)	0.32433 (9)	0.0346 (3)	
C7	0.83951 (10)	0.5554 (2)	0.37348 (11)	0.0455 (4)	
C8	0.88410 (10)	0.6520 (2)	0.42157 (11)	0.0474 (4)	
C9	0.96489 (10)	0.6414 (2)	0.42151 (9)	0.0404 (4)	
C10	0.99748 (9)	0.5262 (2)	0.37327 (10)	0.0378 (3)	
C11	0.73848 (11)	0.0947 (3)	0.06809 (12)	0.0571 (5)	
C12	1.01596 (15)	0.7508 (2)	0.46878 (14)	0.0526 (5)	
N1	0.88262 (7)	0.26572 (15)	0.21515 (8)	0.0341 (3)	
N2	0.95560 (7)	0.43102 (16)	0.32590 (7)	0.0339 (3)	
N3	1.02626 (9)	0.08562 (18)	0.16354 (8)	0.0434 (3)	
N4	1.08684 (8)	0.07905 (18)	0.12842 (8)	0.0402 (3)	
N5	1.14499 (9)	0.0716 (3)	0.09306 (11)	0.0655 (5)	
O19	0.9708 (3)	0.7988 (4)	0.2398 (3)	0.0853 (15)	0.50
H1	0.8876 (10)	0.120 (2)	0.1214 (11)	0.044 (5)*	
H3	0.6660 (14)	0.261 (2)	0.1832 (13)	0.053 (6)*	
H4	0.7209 (11)	0.404 (2)	0.2906 (12)	0.053 (6)*	
H7	0.7824 (12)	0.563 (2)	0.3731 (11)	0.049 (5)*	
H8	0.8591 (13)	0.735 (2)	0.4557 (14)	0.054 (6)*	
H10	1.0548 (12)	0.509 (2)	0.3715 (11)	0.045 (5)*	
H11A	0.7797	0.0357	0.0432	0.086*	
H11B	0.7175	0.1683	0.0302	0.086*	
H11C	0.6978	0.0247	0.0853	0.086*	
H12A	1.0385	0.8272	0.4334	0.079*	
H12B	1.0570	0.6922	0.4944	0.079*	
H12C	0.9850	0.8028	0.5088	0.079*	

### Atomic displacement parameters (Å^2^)

**Table:**

	*U*^11^	*U*^22^	*U*^33^	*U*^12^	*U*^13^	*U*^23^
Ni1	0.02557 (16)	0.03435 (17)	0.03144 (16)	0.000	0.00161 (9)	0.000
C1	0.0336 (7)	0.0443 (9)	0.0403 (8)	−0.0033 (7)	−0.0006 (6)	−0.0012 (7)
C2	0.0361 (8)	0.0476 (10)	0.0455 (8)	−0.0063 (7)	−0.0043 (6)	0.0019 (8)
C3	0.0302 (8)	0.0605 (12)	0.0549 (10)	−0.0022 (7)	−0.0036 (7)	0.0015 (8)
C4	0.0294 (7)	0.0546 (11)	0.0492 (9)	0.0034 (7)	0.0021 (6)	−0.0002 (8)
C5	0.0292 (7)	0.0377 (8)	0.0384 (7)	−0.0002 (6)	0.0018 (5)	0.0033 (6)
C6	0.0304 (7)	0.0378 (8)	0.0354 (7)	0.0030 (6)	0.0020 (5)	0.0024 (6)
C7	0.0369 (8)	0.0533 (11)	0.0462 (9)	0.0066 (7)	0.0046 (7)	−0.0081 (8)
C8	0.0483 (9)	0.0506 (11)	0.0433 (9)	0.0086 (8)	0.0059 (7)	−0.0100 (8)
C9	0.0477 (9)	0.0399 (9)	0.0336 (7)	−0.0002 (7)	−0.0016 (6)	−0.0010 (7)
C10	0.0342 (7)	0.0411 (8)	0.0381 (8)	−0.0004 (6)	−0.0013 (5)	−0.0009 (7)
C11	0.0462 (10)	0.0693 (14)	0.0556 (11)	−0.0115 (9)	−0.0091 (8)	−0.0088 (10)
C12	0.0645 (12)	0.0489 (11)	0.0444 (10)	−0.0032 (8)	−0.0044 (9)	−0.0116 (8)
N1	0.0281 (6)	0.0384 (7)	0.0360 (7)	−0.0014 (5)	0.0007 (5)	0.0000 (5)
N2	0.0313 (6)	0.0357 (7)	0.0347 (6)	0.0013 (5)	0.0017 (4)	−0.0005 (5)
N3	0.0424 (7)	0.0447 (8)	0.0431 (7)	−0.0017 (6)	0.0084 (6)	−0.0076 (6)
N4	0.0373 (7)	0.0448 (8)	0.0383 (7)	0.0076 (6)	−0.0032 (5)	−0.0040 (6)
N5	0.0386 (8)	0.0938 (15)	0.0640 (10)	0.0137 (9)	0.0085 (7)	−0.0099 (10)
O19	0.132 (5)	0.0521 (16)	0.072 (3)	−0.021 (2)	0.026 (3)	−0.0070 (18)

### Geometric parameters (Å, °)

**Table:**

Ni1—N1^i^	2.0882 (13)	C6—C7	1.389 (2)
Ni1—N1	2.0882 (13)	C7—C8	1.379 (3)
Ni1—N2^i^	2.0897 (13)	C7—H7	0.978 (19)
Ni1—N2	2.0897 (13)	C8—C9	1.383 (2)
Ni1—N3	2.1053 (14)	C8—H8	1.00 (2)
Ni1—N3^i^	2.1053 (14)	C9—C10	1.387 (2)
C1—N1	1.340 (2)	C9—C12	1.501 (3)
C1—C2	1.389 (2)	C10—N2	1.340 (2)
C1—H1	1.008 (19)	C10—H10	0.99 (2)
C2—C3	1.392 (3)	C11—H11A	0.9600
C2—C11	1.502 (3)	C11—H11B	0.9600
C3—C4	1.377 (3)	C11—H11C	0.9600
C3—H3	0.98 (2)	C12—H12A	0.9600
C4—C5	1.393 (2)	C12—H12B	0.9600
C4—H4	0.96 (2)	C12—H12C	0.9600
C5—N1	1.347 (2)	N3—N4	1.1901 (19)
C5—C6	1.481 (2)	N4—N5	1.157 (2)
C6—N2	1.3512 (18)	O19—O19^i^	1.053 (9)
			
N1^i^—Ni1—N1	176.19 (7)	C8—C7—C6	119.07 (15)
N1^i^—Ni1—N2^i^	78.26 (5)	C8—C7—H7	121.0 (12)
N1—Ni1—N2^i^	98.99 (5)	C6—C7—H7	119.9 (12)
N1^i^—Ni1—N2	98.99 (5)	C7—C8—C9	120.74 (16)
N1—Ni1—N2	78.26 (5)	C7—C8—H8	121.0 (13)
N2^i^—Ni1—N2	89.80 (7)	C9—C8—H8	118.2 (13)
N1^i^—Ni1—N3	90.53 (5)	C8—C9—C10	116.59 (16)
N1—Ni1—N3	92.13 (5)	C8—C9—C12	122.57 (17)
N2^i^—Ni1—N3	90.12 (6)	C10—C9—C12	120.81 (17)
N2—Ni1—N3	170.26 (5)	N2—C10—C9	123.88 (15)
N1^i^—Ni1—N3^i^	92.13 (5)	N2—C10—H10	114.9 (11)
N1—Ni1—N3^i^	90.53 (5)	C9—C10—H10	121.2 (11)
N2^i^—Ni1—N3^i^	170.26 (5)	C2—C11—H11A	109.5
N2—Ni1—N3^i^	90.12 (6)	C2—C11—H11B	109.5
N3—Ni1—N3^i^	91.61 (8)	H11A—C11—H11B	109.5
N1—C1—C2	123.57 (16)	C2—C11—H11C	109.5
N1—C1—H1	116.1 (10)	H11A—C11—H11C	109.5
C2—C1—H1	120.3 (10)	H11B—C11—H11C	109.5
C1—C2—C3	116.64 (16)	C9—C12—H12A	109.5
C1—C2—C11	120.97 (17)	C9—C12—H12B	109.5
C3—C2—C11	122.39 (16)	H12A—C12—H12B	109.5
C4—C3—C2	120.49 (16)	C9—C12—H12C	109.5
C4—C3—H3	121.3 (12)	H12A—C12—H12C	109.5
C2—C3—H3	118.2 (12)	H12B—C12—H12C	109.5
C3—C4—C5	119.18 (17)	C1—N1—C5	119.11 (14)
C3—C4—H4	119.4 (12)	C1—N1—Ni1	125.82 (11)
C5—C4—H4	121.4 (12)	C5—N1—Ni1	114.73 (10)
N1—C5—C4	120.87 (15)	C10—N2—C6	118.67 (13)
N1—C5—C6	115.47 (13)	C10—N2—Ni1	126.35 (10)
C4—C5—C6	123.61 (15)	C6—N2—Ni1	114.97 (10)
N2—C6—C7	120.95 (15)	N4—N3—Ni1	123.70 (12)
N2—C6—C5	115.17 (13)	N5—N4—N3	178.73 (18)
C7—C6—C5	123.77 (14)		
			
N1—C1—C2—C3	−3.3 (3)	N2—Ni1—N1—C1	176.04 (14)
N1—C1—C2—C11	176.43 (18)	N3—Ni1—N1—C1	−2.32 (14)
C1—C2—C3—C4	3.1 (3)	N3^i^—Ni1—N1—C1	−93.95 (14)
C11—C2—C3—C4	−176.62 (19)	N2^i^—Ni1—N1—C5	−98.70 (11)
C2—C3—C4—C5	−0.2 (3)	N2—Ni1—N1—C5	−10.79 (11)
C3—C4—C5—N1	−2.9 (3)	N3—Ni1—N1—C5	170.84 (11)
C3—C4—C5—C6	174.78 (17)	N3^i^—Ni1—N1—C5	79.22 (12)
N1—C5—C6—N2	−4.0 (2)	C9—C10—N2—C6	0.2 (2)
C4—C5—C6—N2	178.28 (16)	C9—C10—N2—Ni1	178.82 (12)
N1—C5—C6—C7	172.35 (16)	C7—C6—N2—C10	−3.0 (2)
C4—C5—C6—C7	−5.4 (3)	C5—C6—N2—C10	173.45 (14)
N2—C6—C7—C8	3.1 (3)	C7—C6—N2—Ni1	178.23 (13)
C5—C6—C7—C8	−173.01 (16)	C5—C6—N2—Ni1	−5.34 (17)
C6—C7—C8—C9	−0.4 (3)	N1^i^—Ni1—N2—C10	7.22 (14)
C7—C8—C9—C10	−2.2 (3)	N1—Ni1—N2—C10	−170.09 (14)
C7—C8—C9—C12	175.99 (19)	N2^i^—Ni1—N2—C10	−70.86 (13)
C8—C9—C10—N2	2.4 (3)	N3^i^—Ni1—N2—C10	99.40 (14)
C12—C9—C10—N2	−175.83 (16)	N1^i^—Ni1—N2—C6	−174.10 (10)
C2—C1—N1—C5	0.4 (3)	N1—Ni1—N2—C6	8.59 (10)
C2—C1—N1—Ni1	173.26 (13)	N2^i^—Ni1—N2—C6	107.82 (11)
C4—C5—N1—C1	2.8 (2)	N3^i^—Ni1—N2—C6	−81.92 (11)
C6—C5—N1—C1	−175.05 (14)	N1^i^—Ni1—N3—N4	−32.56 (15)
C4—C5—N1—Ni1	−170.89 (13)	N1—Ni1—N3—N4	144.70 (14)
C6—C5—N1—Ni1	11.29 (17)	N2^i^—Ni1—N3—N4	45.70 (14)
N2^i^—Ni1—N1—C1	88.14 (14)	N3^i^—Ni1—N3—N4	−124.71 (16)

Symmetry codes: (i) −*x*+2, *y*, −*z*+1/2.
